# The Prenatal Diagnosis and Perinatal Management of Congenital Long QT Syndrome: A Comprehensive Literature Review and Recent Updates

**DOI:** 10.3390/jcdd12040156

**Published:** 2025-04-14

**Authors:** Stefani Samples, Sara Cherny, Nitin Madan, Jeff Hong, Sheena A. Mansukhani, Janette F. Strasburger, Michael R. Carr, Sheetal R. Patel

**Affiliations:** 1 Division of Pediatric Cardiology, Ann & Robert H. Lurie Children’s Hospital of Chicago, Northwestern University Feinberg School of Medicine, Chicago, IL 60611, USA; scherny@luriechildrens.org (S.C.); jehong@luriechildrens.org (J.H.); smansukhani@luriechildrens.org (S.A.M.); mcarr@luriechildrens.org (M.R.C.); spatel@luriechildrens.org (S.R.P.); 2 Division of Pediatric Cardiology, Children’s Mercy Kansas City, Ward Family Heart Center, UMKC School of Medicine, Kansas City, MO 64108, USA; nmadan@cmh.edu; 3 Division of Cardiology, Department of Pediatrics and Biomedical Engineering, Children’s Wisconsin, Herma Heart Institute, Medical College of Wisconsin, Milwaukee, WI 26509, USA; jstrasburger@childrenswi.org

**Keywords:** fetal echocardiography, arrhythmia, genetics, inherited arrhythmia syndrome, fetal magnetocardiography, electrophysiology

## Abstract

Congenital long QT syndrome (LQTS) is a group of heritable conditions that are associated with cardiac repolarization abnormalities characterized by QT prolongation on electrocardiogram and the risk of life-threatening arrhythmias. The prenatal detection of LQTS presents significant challenges for clinicians, and a multidisciplinary approach is required for optimal prenatal and postnatal management. In this comprehensive literature review, we describe strategies for the fetal diagnosis of LQTS with variable initial presentation, genetic testing in suspected fetal LQTS, the utility of fetal magnetocardiography as an additional diagnostic tool, prenatal management, and postnatal treatment. We focus on a multidisciplinary team approach including fetal cardiology, adult and pediatric electrophysiology, neonatology, maternal–fetal medicine, and genetic counselors, all playing vital roles in the comprehensive prenatal management and orchestration of postnatal treatment to optimize neonatal outcomes.

## 1. Introduction

Long QT syndrome (LQTS) is a group of conditions described as ion channelopathies characterized by the prolongation of the QT interval on a surface electrocardiogram (ECG) due to abnormalities in repolarization. It carries an associated risk for life-threatening ventricular arrhythmias that can result in intrauterine fetal demise, cardiac arrest, and sudden cardiac death at any age [1,2]. LQTS is a genetic condition that is either inherited or presents as a *de novo* mutation [2]. The estimated prevalence of LQTS based on genetic testing and ECG findings is at least 1 in 2500 healthy white live births and may be as high as 1 in 1000 based on ECG-determined prolonged QTc (QTc > 450 ms) during early infancy [3,4]. Since sudden cardiac death is more likely to be the initial present event in children, prompt diagnosis and initiation of treatment for LQTS is important [5].

Fetal arrhythmias complicate approximately 1–3% of pregnancies and can result in significant morbidity and mortality for the fetus. LQTS is thought to be the potential cause for up to 10% of fetal demises, in utero hydrops, and premature deliveries [6]. There is an 8.8% incidence of genetic variants leading to dysfunctional LQTS-associated ion channels in vitro in stillborn infants, according to a study reviewing 91 unexplained intrauterine fetal deaths [7]. *De novo* LQTS variants are particularly high-risk for lethal complications and a significant cause of stillbirth [1,8]. Given this level of fetal and neonatal morbidity and mortality seen with LQTS, early diagnosis and treatment is critical. In this paper, we will review the prenatal presentation, diagnosis, and management of long QT syndrome, with a particular focus on available diagnostic modalities, genetic testing considerations, fetal management strategies, delivery planning, and perinatal care.

## 2. Fetal Diagnosis of Long QT Syndrome

Fetal LQTS diagnosis can be challenging given the variable presentations and limitations in diagnostic testing during fetal life. It requires a high index of suspicion based upon fetal imaging and related diagnostic studies, as well as maternal and family medical histories. Fetal echocardiography is the main diagnostic modality for fetal LQTS given its wide availability in most centers. However, this modality does not have the ability to directly measure the QT interval. Instead, the suggestion of LQTS is made through the identification of one or more of the associated characteristic rhythms, including fetal sinus bradycardia, 2:1 atrioventricular (AV) block, and ventricular arrhythmias.

Fetal sinus bradycardia has historically been defined as fetal heart rate < 110 beats per minute (bpm) at any gestational age. Sinus bradycardia can be normal if secondary to increased vagal tone in the fetus, as occurs with pressure from the ultrasound probe during imaging, or with the use of certain maternal medications which can have negative fetal chronotropic effects, but it can also be a concerning sign of fetal distress or pathologic fetal arrhythmia. The presence of a low fetal heart rate (FHR) has long been associated with potential LQTS [5,9]. More recent studies have noted that repeated FHR measurements less than the third percentile for gestational age can be an indication of inherited arrhythmia syndromes such as LQTS and may be a more useful cutoff than the historical cutoff of 110 bpm for the purposes of clinical management [10,11]. Another indication of fetal LQTS identifiable on fetal echocardiography is a prolonged left ventricular isovolumetric relaxation time (LVIRT). During the fetal life, LVIRT is estimated using left ventricular inflow and outflow pulsed-wave Doppler wave forms and measuring the time between the end of the aortic ejection wave and the beginning of the mitral inflow wave form. Clur et al. demonstrated that LVIRT was prolonged in all gestational age groups with LQTS, and their recommended diagnostic cutoff for LQTS was a normalized LVIRT greater than 11.3 at or before 20 weeks gestation [12]. Utilizing both fetal bradycardia and LVIRT can enhance the detection of LQTS by fetal echocardiography. Phan et al. demonstrated a strong direct correlation between the length of the QTc and the LVIRT using fetal magnetocardiography (fMCG) [13].

**Figure 1:**
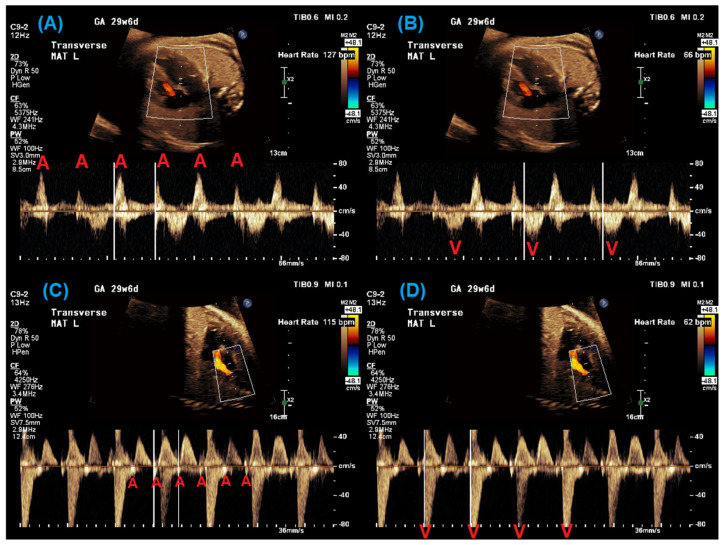
Pulsed-wave Doppler evaluation of a 29-week fetal patient with 2:1 AV block. From left to right: The evaluation of mitral valve inflow–aortic valve outflow Doppler with atrial rate (**A**) and ventricular rate (**B**) measured and superior vena cava–aorta Doppler with atrial rate (**C**) and ventricular rate (**D**) measured. Red A = atrial beat; red V = ventricular beat.

**Figure 2:**
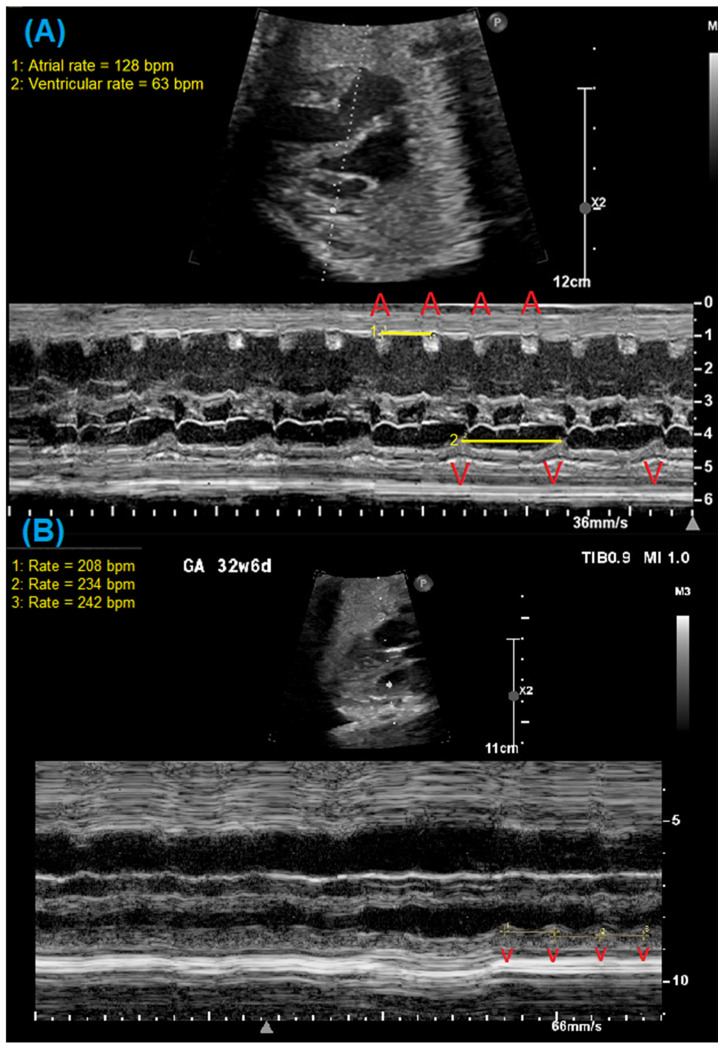
M-mode evaluation of two fetal patients with confirmed LQTS. (**A**) M-mode cursor through atria and ventricular walls demonstrating 2:1 AV block in a 29-week gestational age fetus. (**B**) M-mode of short episode of ventricular tachycardia with ventricular rate > 200 bpm in a 32-week gestational age fetus. Red A = atrial beat; red V = ventricular beat.

Other common abnormal rhythms associated with fetal LQTS include 2:1 AV block and ventricular arrhythmias, particularly Torsades de Pointes (TdP) [1]. Bradycardia due to 2:1 AV block can be identified by evaluating the timing and relationship between the atrial and ventricular contractions using simultaneous pulsed-wave Doppler interrogation of the left ventricular inflow and outflow (e.g., mitral valve–aortic valve), the superior vena cava and aorta, or a pulmonary vein and pulmonary artery [14]. Figure 1 demonstrates examples of such Doppler waveforms in a fetal patient with 2:1 AV block. M-mode imaging of the atrial and ventricular walls can also identify 2:1 AV block as well as ventricular arrhythmias. Figure 2 demonstrates m-mode evaluation in two fetal patients with 2:1 AV block and a short, captured episode of ventricular tachycardia. The identification of ventricular arrhythmias can be challenging as they may only occur intermittently, stressing the need for more prolonged assessment during fetal echocardiography when arrhythmia is suspected. Even with methodical imaging, diagnosis can be elusive. Wacker et al. and Strand et al. have reported patterns of irregular valve clicks during TdP without associated aortic flow velocities and the presence of “normal-rate” TdP, making it difficult to identify TdP even when present, because the Doppler pattern does not mimic a typical tachycardia [8,15].

It is important to understand that all the above-described methods for fetal cardiac rhythm assessment extrapolate or estimate electrical signaling based on mechanical events in the fetal heart. For more direct measurements of the electrical signals and QT interval in the fetus, more advanced modalities are necessary. Noninvasive fetal electrocardiography (NIFECG) is more readily available, as there are several devices approved by the Food and Drug Administration (FDA) [16]. However, NIFECG has primarily been utilized for FHR detection, particularly during labor, and tracings can be complicated by other sources of interference even after adequate maternal ECG suppression [17]. Therefore, the utility of NIFECG in accurately measuring QTc and its use in early gestational period have not yet been validated. Additionally, research is underway to develop techniques for obtaining a fetal electrocardiogram (fECG). Sethi et al. evaluated three cases of suspected LQTS and used fECG technology based on blind source separation with a reference signal, and the fECG findings corroborated the diagnosis of LQTS [18]. Such technology may provide a more cost-effective and readily available diagnostic modality but needs to be further developed and validated. Another limitation in improving the applicability of these fetal ECG technologies to routine clinical practice is the amount of time and skill required for the post processing of the data to generate accurate clinical information. However, this limitation may be overcome by using artificial intelligence and machine learning technology. For example, a recent retrospective observational study measured cardiac time intervals of the fECG utilizing a computerized algorithm to analyze archived ECGs performed using a MonicaAN24 monitor and with a suitable signal-to-noise ratio [19]. The comparison of manually calculated intervals was similar to the computer-generated measurements, indicating that such computer algorithms can help further develop fECG technologies to make it more easily available, faster, and cost effective. Fetal magnetocardiography is another advanced modality that, while not as readily available, adds significantly to the diagnosis of fetal LQTS [20].

## 3. Utility of Fetal Magnetocardiography

Fetal magnetocardiography is an FDA-approved technology that uses sensitive biomagnetometers based on superconducting quantum interference device (SQUID) passive magnetometers without emitting energy or magnetic fields [21]. Fetal magnetocardiography has the potential to more accurately diagnose fetal arrhythmias and reduce diagnostic ambiguity by evaluating depolarization and repolarization through cardiac intervals (RR, P, PR, QRS, WT, U and QTc), signal characteristics, and unique rhythm patterns, similar to postnatal ECG [20,22]. The utility of fMCG is best for fetuses over 24 weeks’ gestation, though it does have some utility in fetuses from 17 to 24 weeks’ gestation as well [21].

**Figure 3:**
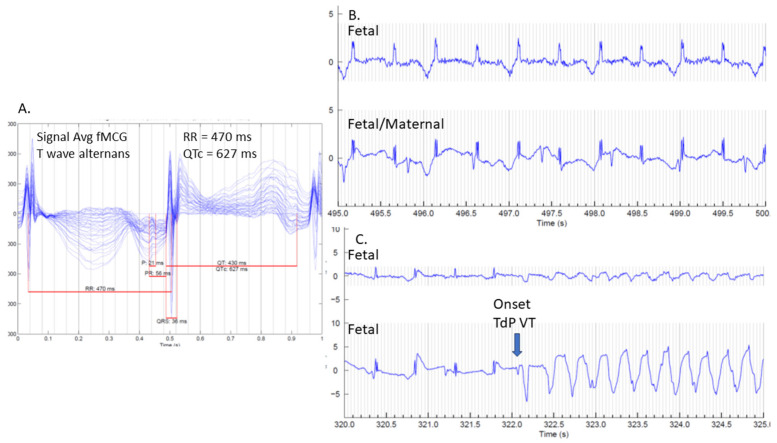
The fMCG evaluation of a 36-week gestational age fetus with familial LQT 2: (**A**) Signal-averaged fMCG showing 3 QRS complexes. Note that the T waves are of different morphology and polarity as well as very prolonged (627 ms QTc). (**B**) A rhythm strip showing A:B:A:B-type T wave alternans during 1:1 AV conduction. Maternal complexes can also be seen in the lower panel. (**C**) Five-second rhythm tracing, showing the onset of organized TdP ventricular tachycardia, which at the time exceeded 300 beats per min.

**Figure 4:**
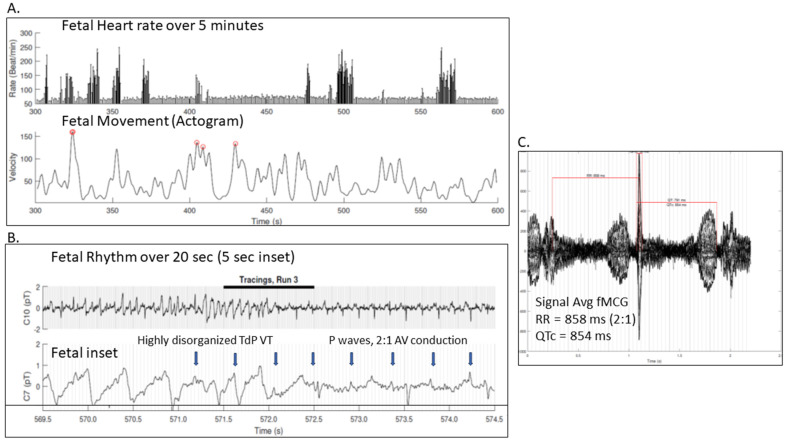
The fMCG evaluation of a 26-week gestational age fetus with negative family history, 2:1 AV block, and hydrops fetalis: (**A**) Fetal heart rate trend graph and fetal movement (actogram) over 5 min, showing multiple brief episodes of fetal rhythm a little faster than sinus, yet the pattern is due to TdP. This fetus had spontaneous fetal demise at 28 weeks gestational age and later was confirmed with genetics to have *de novo* LQT3, SCN5A mutation. (**B**) 20 s and 5 s rhythm tracings showing disorganized TdP ventricular tachycardia self-terminating to 2:1 AV block, with severe QTc prolongation. (**C**) Signal-averaged fMCG showing central QRS with severe QTc prolongation 854 ms. This fetus did not show prominent T wave alternans.

As the only modality able to identify otherwise hidden depolarization and repolarization abnormalities, fMCG is the most accurate and comprehensive method for diagnosing a prolonged QT interval in a fetus presenting with a familial arrhythmia risk or fetal arrhythmias [1,6,8,20,23,24]. It has over 90% sensitivity and specificity for the diagnosis of LQTS [15]. Figure 3 demonstrates fMCG results on a 36-week fetus with familial LQTS type 2 and captured TdP during the study. Fetal magnetocardiography also allows care teams to more accurately diagnose LQTS in fetuses that present with complex tachy–brady arrhythmias, where fetal echocardiography is non-diagnostic or ambiguous. It also provides additional confirmation in challenging cases without a family history of LQTS. Figure 4 demonstrates fMCG findings in a 26-week fetus without a family history of LQTS but severe QT prolongation by fMCG.

A 10-year retrospective review published in 2022 by Wacker-Gussmann et al. demonstrated that fMCG has the capacity to alter or expand the fetal diagnosis. In this study of 144 fetuses, fMCG altered or expanded the fetal diagnoses in 117 fetuses, with 81 of them undergoing a “clinically important” change in diagnosis, and fMCG altered management in 76% of cases (109/144) [20]. Of interest, LQTS presented in all categories (fetal tachycardia, sinus bradycardia, AV block, and family history). A total of 16 fetuses presenting with either tachycardia or sustained bradycardia had a change in diagnosis to prolonged QTc. Of the 39 fetuses with a familial LQTS risk, 25 (64%) were found to have LQTS via fMCG (with only 1 false-positive confirmed postnatally). This demonstrates the invaluable role of fMCG in fetal arrhythmia assessment and the importance of correct fetal rhythm diagnosis in potentially improving maternal–fetal outcomes [20,24]. The benefit of a prenatal diagnosis of LQTS is substantial and allows for intrauterine treatment, the potential avoidance of preterm delivery, risk reduction with the maternal avoidance of QTc prolonging medications, the prevention of fetal and perinatal TdP, the anticipation of postnatal arrhythmia concerns, and the earlier identification of affected but asymptomatic family members [6,25].

## 4. Genetics

### 4.1. Genetics of LQTS

Long QT syndrome is perhaps the most well described of the genetic arrhythmia syndromes. The majority of individuals with LQTS have a variant in one of three genes: KCNQ1, KCNH2, and SCN5A, comprising LQTS types 1, 2, and 3, respectively [26]. Beyond these three genes, the genetic underpinnings of LQTS begin to overlap with other syndromes [27]. For example, CACNA1C is associated with a spectrum of cardiac phenotypes including not only LQTS but also Timothy syndrome, characterized by syndactyly, neurodevelopmental findings, and a high risk of arrhythmia events in the neonatal period [28]. KCNJ2 is associated with Andersen–Tawil syndrome, which includes a number of extracardiac features such as episodes of paralysis and muscle weakness [29]. Knowledge of the calmodulin genes (CALM1, CALM2, CALM3) is emerging and suggests that calmodulinopathy, while similar to LQTS, is in fact a distinct entity likely to affect children early in life [30,31]. On the less severe end of the LQTS spectrum, KCNE1 and KCNE2 are associated with acquired prolonged QT, with variants that are relatively common in the population [32]. That said, variants in these genes may be reported in more severe presentations, such as fetal arrhythmia or even stillbirth. It is important for providers to remain as current as possible regarding the range of possible phenotypes and genotype–phenotype correlations for genes in the LQTS spectrum [11]. As our knowledge about the genetic etiologies of prolonged QT improves, this list of genes will continue to evolve. To best inform clinical decision making in the fetal–neonatal period, a provider with expertise in the genetics of LQTS should be included in the care team [33].

It has been consistently estimated for over a decade that molecular genetic testing identifies a pathogenic variant in over 75% of individuals with a clinical diagnosis of LQTS [34]. Since many forms of LQTS are associated with autosomal dominant inheritance, most gene-positive individuals will have one variant in one gene detected. There is a meaningful rate of *de novo* variants for individuals with LQTS; therefore, the lack of a family history does not rule out the possibility of a genetic etiology. Furthermore, LQTS exhibits reduced penetrance and variable expressivity, both within and between families. Various forms of LQTS are frequent enough in the general population that it is reasonable to expect the occasional family to have a variant in both copies of one LQTS gene (biallelic) or a digenic pattern of disease in which there are two separate forms of LQTS in the family [35,36,37,38]. These genotypes are likely to result in more severe phenotypes, such as Jervell and Lange-Nielsen syndrome, due to variants in both copies of either the KCNQ1 or KCNE1 gene, and are associated with congenital bilateral hearing loss and QTc intervals over 500 ms [39].

### 4.2. Genetic Testing Approaches

Genetic testing is a critical contributor to diagnosis for LQTS. Since there are clear genotype–phenotype associations for many forms of LQTS, genetic testing can help confirm the clinical diagnosis, guide management, and provide prognostic information [40,41].

Multi-gene panel tests are the norm for new diagnosis of suspected LQTS [33]. Panel tests are efficient, thorough, and cost-effective. They may be phenotype-specific; for example, panels may cover genes associated primarily with LQTS. Alternatively, panels may include a broad list of genes associated with arrhythmias and cardiomyopathy syndromes that may be of most use when the clinical presentation is unusual or uncertain. On the other hand, patients in the fetal cardiology setting may have been referred due to a history of LQTS in a parent who may or may not have had genetic testing themselves. If there is a known variant in the family, and prenatal testing is desired, it is usually only necessary to test for the familial variant. If a familial variant has not yet been identified, the affected parent or sibling should have genetic testing to determine if a variant is detectable, and then testing for only the familial variant(s) is warranted for the pregnancy or neonate [40,42]. Given the wide variety of commercial laboratories, as well as the ever-changing nature of the market, we recommend seeking advice from a genetic counselor regarding test type and methodology, as well as choice of testing lab [43]. Most LQTS-related variants are detectable on sequencing. However, it is also possible to identify variants on other testing modalities, such as a chromosome microarray which detects whether there is genetic material that is duplicated or deleted. However, it is important to recognize that a reportedly normal chromosome microarray will not exclude all genetic etiologies of LQTS as would be detected on a LQTS gene panel. The involvement of genetic counselors is vital to see that the proper test is ordered in an era of ever-increasing complexity of testing [42,43].

The possibility of parental mosaicism should be considered in some families. Mosaicism is defined as the presence of two or more cell lines in one individual. For example, gonadal mosaicism occurs when a parent has a genetic variant only in the reproductive organs, such that they will not present with a phenotype but will have the risk of passing the variant on to a child. Situations to consider mosaicism include multiple fetal losses or when more than one sibling is diagnosed with clinical LQTS while the parents have apparently negative genetic testing [44]. Additional parental specimens such as skin biopsy, semen, urine, and saliva may identify the variant and confirm the presence of mosaicism.

There are few publications regarding prenatal diagnostic genetic testing for LQTS [45,46]. Recent work from Killen and Strasburger suggests obtaining postnatal genetic testing in fetal arrhythmia cases [6]. Chivers et al. note two cases where prenatal diagnostic genetic testing had an impact on management [1]. The decision about whether to have prenatal diagnostic testing must prioritize patient informed consent, and include discussion of risks, benefits, and limitations as well as parent reproductive goals [47]. The timing of elective genetic testing in the perinatal setting is an important consideration for any pregnancy. The timing of the prenatal diagnostic test must be aligned with parent preferences regarding continuation of the pregnancy. Genetic testing can be conducted as early as 10–12 weeks of pregnancy via chorionic villus sampling (CVS). Amniocentesis can be conducted from around 16 weeks of pregnancy throughout the remainder of gestation. Further, the choice of procedure must be balanced with the risk of miscarriage (1% or less for CVS, 1/500 or less for amniocentesis) and each parent may have a different risk tolerance as they consider the potential value of the genetic test result. More specifically in the context of a fetus with or at risk for arrhythmia, preterm labor resulting from an amniocentesis after the age of viability could result in the delivery of a preterm infant who will need complex neonatal care in addition to potential electrophysiological intervention. Thus, in the case of fetal LQTS, the benefits of amniocentesis later in a pregnancy may not outweigh the risk. If management and treatment recommendations can be made based on clinical findings, especially with the use of fMCG, it may be reasonable to postpone genetic testing until after delivery. Given the multiple considerations, thorough counseling and shared decision making with patients should be used for a patient-specific determination of care pathway.

### 4.3. Genetic Test Results

In general, genetic testing results return in one of three ways: positive, negative, and uncertain. A positive result is one that identifies one or more clearly pathogenic variants in a gene that is associated with the clinical phenotype in question, i.e., LQTS. Especially in the prenatal setting, a positive genetic test does not constitute a diagnosis of LQTS. While there is a tendency for more severe forms of LQTS to present prenatally, all types of LQTS exhibit reduced penetrance and variable expressivity; some individuals with a pathogenic variant will never present with features of LQTS, and those that do will vary in age of onset, severity, and presentation. Although the yield of panel-based genetic testing for LQTS is high when compared to other arrhythmia syndromes, it does not yet approach 100%. Due to this limited sensitivity, a negative result cannot rule out the possibility of an underlying genetic contribution to the patient’s phenotype. However, a negative result in the context of a known pathogenic familial variant can usually be considered a true negative, with the caveat of the possibility of multiple genetic variants in a family. It is theoretically possible that a fetus may have a phenotype that is unique from their parent with LQTS. For example, Phan et al. showed that even with normal fMCG, fetuses with familial LQTS had intermediate LVIVRT measurements compared to those normal subjects without a family history [13]. It is important to view each case independently without assumption of outcome or test result.

Variants of uncertain significance (VUS) are very common in cardiovascular genetic testing and may account for up to 75% of arrhythmia and cardiomyopathy genetic test results [48,49,50]. Authors have reported the yield of genetic testing in suspected fetal LQTS, but there are no data at this time regarding the frequency of uncertain variants [11]. One way to minimize uncertainty in genetic testing is to limit the number of genes tested [51]. On the other hand, while the majority of variants identified in individuals with LQTS will be identified in a small number of genes, a broader panel may allow for the coverage of genes that confer a phenotype that overlaps with LQTS. Castillo et al. demonstrated that comprehensive genetic testing revealed diagnoses that would have been missed by disease-specific testing [49]. Given the increase in results returned on genetic testing, Waddell-Smith and others emphasize the importance of understanding pre-test probability as well careful phenotyping in result interpretation [52,53]. Most importantly, a variant of uncertain significance cannot be used for clinical decision making [54]. The clinician or multidisciplinary team must rely on their expertise and clinical judgment, and carefully counsel patients and families that a VUS does not confer any information about risk or diagnosis.

One of the most important aspects of genetic testing and results interpretation in current cardiovascular genetics practice is variant adjudication. Variant classification guidelines have been set by the American College of Medical Genetics and Genomics and the practice continues to evolve through careful curation by experts such as the Clinical Genomics Resource [54,55]. Classification criteria relate to factors such as patient phenotype and family history, the de novo status of the variant, published in vitro and in vivo functional studies, animal models, population and cohort studies, family segregation studies, and a computational analysis of the impact of the variant on the gene and protein. There is an allowance for some subjectivity in the guidelines, which can lead to differences in variant classification between laboratories [56]. Differences in variant interpretation can have clinical impact—the classification of a reported variant may affect medical management, cardiac screening recommendations, and family testing recommendations [57]. Further, the importance of ongoing variant reclassification in LQTS and arrhythmia syndromes has been documented, as up to 10–15% of variants may change classification over time in a meaningful way [48,50]. In addition, data have shown that lack of diversity in genetic studies can lead to health disparities with regard to variant classification. Insufficient data regarding variant frequency in minority populations can lead to misclassification of benign variants as clinically significant [58]. As more research and clinical data are gathered about a particular gene or variant, it may be possible to reassess a variant’s classification. Thus, variant classification is an ongoing process, and many variants will remain unclassified for a number of years. Genetic counselors are integral multidisciplinary team members who can lead variant interpretation efforts, and close collaboration between cardiologists, other specialists, and genetic counselors has been consistently recommended [43,45,59,60,61,62].

### 4.4. Postnatal Genetics Considerations

Advances in the quality and coverage of genetic testing in the prenatal setting have led to consistently high-quality testing modalities and accurate testing results. Given the rare possibilities of complex genetic etiologies of LQTS, if the neonatal phenotype does not match the prenatal expectation, additional or confirmatory genetic testing may be warranted in the neonatal period. Family cascade clinical cardiac and genetic testing, however, can begin at any time. Cascade genetic screening is the practice of efficiently and methodically evaluating at-risk family members to identify those who may also be at risk for LQTS. The timely cascade screening of family members can prevent the morbidity and mortality that is associated with LQTS and can allow ample time for a patient’s family unit to consider their plans for the future.

## 5. Fetal Management

Managing the fetus with LQTS involves balancing fetal and maternal therapeutic needs in the setting of overall pregnancy management. Because of this, maternal–fetal medicine (MFM) specialists and adult electrophysiologists play important roles in caring for these mother–fetus dyads. Given the genetic inheritance of LQTS, when it is identified in the fetus, parental testing is warranted clinically with ECGs and the subsequent adult cardiology evaluation of any abnormalities noted. If the mother has LQTS, there are implications for care during pregnancy and delivery. Maternal anesthesia and medication choices may warrant adjustments to avoid QT prolonging agents which may affect the mother as well as the fetus [15]. Maternal calcium, magnesium, and 25-OH-vitamin D levels should also be optimized during pregnancy and delivery [21].

Frequent fetal echocardiograms are warranted when there is suspected LQTS to monitor the fetal heart rate and rhythm, as well as to evaluate for signs of heart block or ventricular arrhythmias such as TdP. In between fetal echocardiography evaluation by professionals, home fetal heart rate monitoring (HFHRM) can assist in detecting abnormally fast or slow FHR using a home hand-held Doppler for daily FHR assessment by parents to detect evolving arrhythmias. HFHRM has already demonstrated success in the early identification of AV block during surveillance of anti-Ro pregnancies [63]. While not all centers have a home Doppler available for patient use and resources to address patient concerns from HFHRM, it can provide additional support to families where these resources are available.

Heart block is relatively uncommon in LQTS types 1 and 2, but more common in SCN5A and rarer variants [8]. When the fetus presents with ventricular arrhythmias, the prompt initiation of anti-arrhythmic therapies can reduce the risk of intrauterine fetal demise [64]. Appropriate medication selection is critical, since some typical treatments of fetal ventricular tachycardia such as sotalol and amiodarone can prolong the QTc interval and increase risk of TdP in fetuses with LQTS. Instead, recommendations for treatment include magnesium and 25-OH-vitamin D supplementation as well as antiarrhythmics such as mexiletine, lidocaine, or propranolol [65,66]. Ongoing MFM monitoring during treatment is needed, as mild fetal growth restriction has been reported with beta-blocker usage [15]. The question sometimes arises as to whether treatment with beta-blockers should be initiated prenatally for confirmed cases, or those with QTc prolongation or IVRT prolongation. At present, using a conservative strategy of only prenatally treating extremely high-risk cases (those with TdP) with beta-blockers, there have been no reports of mortality in familial cases of LQTS, and we would therefore not advise arbitrary prenatal beta-blocker treatment in the absence of high risk. *De novo* cases have had higher risk of fetal mortality, but it is often difficult to be sure that the case is *de novo*, since based on published experiences, many turn out to be familial but not previously diagnosed. Based on fMCG, if LQTS is highly suspected with higher risk for fetal arrhythmia indicated by a fetal sinus rate of less than 120 bpm or 2:1 atrioventricular block, we would strongly consider utilizing HFHRM if available. Such high-risk cases may also benefit from a repeat fMCG obtained at a later gestation as well. Maternal beta-blocker therapy may be considered in cases where the QTc is more than 600 ms with T wave alternans or the presence of late-coupled PVCs. However, no such cases with these potentially high-risk features have yet been encountered without concurrent evidence of TdP, so beta-blocker therapy would already be recommended. In summary, it appears that the risk of routine maternal beta-blocker use for both the mother and fetus outweighs the benefits in LQTS unless high-risk features are present.

Comprehensive prenatal counseling can also aid in parental preparedness for other postnatal care considerations. This can include information on postnatal medical and surgical treatment with potential pacemaker or intracardiac defibrillator placement, which is further detailed below. Other resources and support organizations, such as the Sudden Arrhythmia Death Syndromes Foundation, can provide helpful education on reproductive options, pregnancy preparedness during the “fourth trimester”, CPR education resources, and family-based conferences.

Delivery planning should involve a multi-disciplinary cardio-obstetrics team, particularly in cases that involve maternal LQTS. Continuous ECG monitoring is important during labor, as is delivery at a tertiary care center with appropriate maternal and pediatric subspecialists available [24]. Full-term gestations are expected and typical, in-hospital labor with vaginal delivery is appropriate for fetuses with less severe phenotypes; however, the mode of delivery may require modification if the fetal heart rate and rhythm do not allow for adequate fetal monitoring during labor [15]. For example, approximately one-third of LQTS fetuses have low heart rate reactivity at baseline, and therefore, non-stress testing is not as reliable as biophysical profiles in assessing fetal wellbeing late in gestation. Collaboration between MFM, neonatology, and adult and pediatric cardiology or electrophysiology can ensure appropriate preparation of the delivery room, including resources for acute resuscitation for the affected mother or fetus. Anesthesia should be prepared to alter their standard strategies by avoiding QT prolonging agents and maintaining appropriate maternal electrolyte levels, particularly magnesium [15]. Additional specialists needed in the delivery room or on standby for emergent postnatal procedures such as transvenous or epicardial pacing can be determined based upon risk stratification by fMCG, as well as the fetal echocardiogram findings near delivery [67,68,69].

## 6. Postnatal Management

### 6.1. Delivery Location and Delivery-Room Resource Preparation

Many newborns with LQTS will have an uneventful delivery-room course; nonetheless, delivery of a newborn with LQTS should occur at a hospital with access to cardiac and/or cardiac electrophysiology expertise to help manage any emergent ventricular arrhythmias that arise in the delivery room or neonatal unit. Delivery-room management is based on the prenatal fetal cardiac rhythm indicating risk of neonatal tachyarrhythmia and clinical presentation after birth as described previously. For newborns at high risk of developing ventricular arrhythmia, intravenous access, typically an umbilical venous catheter, should be obtained shortly after delivery, and an external defibrillator should be readily available in the delivery room.

### 6.2. Management of Torsades de Pointes

TdP is a medical emergency. Episodes of TdP can be self-limiting, but sustained runs result in inadequate perfusion and can potentially degenerate into ventricular fibrillation. Intravenous magnesium is used as first-line pharmacologic therapy for TdP, even in patients with normal magnesium levels [70,71,72]. Unstable TdP or ventricular fibrillation requires immediate cardioversion. In addition, sedation, intubation, and neuromuscular blockade may be utilized to minimize adrenergic stimuli. Short-term intravenous beta-blockers along with infusions of magnesium usually stabilize the rhythm, but if needed, other agents such as intravenous lidocaine may be necessary. Rarely, TdP can also manifest as a “normal rate” ventricular tachycardia, which appears almost monomorphic, or triggered by atrial flutter. Fortunately, TdP is not common during the neonatal period for most cases of LQTS, but advanced preparation to manage such an emergency can improve outcomes.

### 6.3. Management of Bradycardia

The most likely ventricular arrhythmia to arise in a newborn with LQTS is sinus bradycardia, especially as one initiates beta-blocker therapy. Most sinus bradycardia is well-tolerated, with stable blood pressure and adequate end-organ perfusion, despite the heart rate being lower than the normal range. For infants with significant bradycardia resulting in hemodynamic instability, the focus should be optimizing oxygen delivery to meet end-organ demand. Such newborns require frequent clinical assessment of oxygen delivery, which can include monitoring lactate level, renal biomarkers, urine output, renal and/or cerebral near-infrared spectroscopy, respiratory effort, and mental status. Hemodynamically significant bradycardia or bradycardia/pause-induced ventricular arrhythmias may require emergent pacing [15]. In a recent review by Chivers et al., 34 of 61 of neonatal LQTS cases required pacing. It is unclear to what extent cardiac pacing confers a long-term reduction in risk over that of beta-blockers, but in the setting of needs for beta-blocker therapy, pacing is often employed to maintain heart rate.

Emergency temporary pacing can be instituted via a transvenous approach from the umbilical, femoral, or internal jugular vein at a center with cardiac electrophysiology expertise. Alternatively, temporary or permanent pacing via an epicardial approach can be performed by a cardiac surgeon. Given the relatively large size of pacing leads and pacemaker generators, avoidance of preterm delivery is preferred.

### 6.4. Antiarrhythmic Therapy for Long-Term Management

After the initial stabilization of the cardiac rhythm, beta-blockers are the cornerstone maintenance medical therapy for neonates with LQTS, as their anti-adrenergic effect is critical in reducing sudden cardiac events among the three major genotypes of LQTS (types 1, 2, and 3). In several registry-based studies, the use of a beta-blocker has been associated with a 50–70% reduction in risk of a sudden cardiac event [73,74,75,76]. The nonselective beta-blockers propranolol and nadolol have shown higher efficacy than their selective counterparts [77,78,79]. Propranolol has also been shown to block late sodium current in in vitro models, explaining its effect in reducing the QT interval clinically [77,80,81]. In addition to beta-blockers, sodium-channel blockers may be used; amongst different sodium channel blockers, the use of mexiletine in LQTS type 3 has been the most extensively studied. As LQTS type 3 is caused by a gain-of-function mutation in the SCN5A gene, the use of mexiletine as a gene-specific therapeutic strategy has been shown to significantly decrease the QT interval and the risk of an arrhythmic event [82,83,84]. To a lesser extent, the successful use of flecainide to shorten the QT interval in LQTS type 3 has also been reported [85,86]. Even in patients without sodium-channel mutations, mexiletine can be considered if the QT interval is markedly prolonged, as it has been shown to shorten QT intervals in subsets of patients with potassium-channel-mediated LQTS [87]. Finally, for mothers who are breastfeeding, small amounts of lipid-soluble beta-blockers such as nadolol may transfer through breastmilk and augment the beta-blockade effect in their newborn, reinforcing the fact that the maternal–fetal dyad does not necessarily cease to exist after delivery.

### 6.5. Pacemaker of Intracardiac Defibrillator Insertion for Long-Term Management

In patients with persistent bradycardia- or pause-induced ventricular ectopy or arrhythmias despite treatment with beta blockade and mexiletine, permanent pacing may be indicated and has been shown to prevent the bradycardia-related triggering of arrhythmias in neonates [2,68,88,89]. In contrast to pacemaker implantation, there is significantly less data on implantable cardioverter–defibrillators (ICD) in newborns. Implanting an ICD in a neonate has been reported, though it often requires non-standard approaches and can carry significant morbidities. In addition to the typical known risks associated with an ICD, including infection, lead failure, and inappropriate shocks, there are also additional risks likely related to the size mismatch of implanting a relatively large device in a small neonate, such as chylothorax and feeding intolerance [90,91,92]. In medium- to long-term follow-up studies, patients with LQTS type 2 or type 3 and patients with *de novo* LQTS mutations are more likely to suffer sudden cardiac events and to require permanent pacing or cardioverter–defibrillator therapy [1,93]. For high-risk neonates and infants with LQTS, an alternative to primary prevention ICD can consist of optimizing medical therapy, considering left cardiac sympathetic denervation, and recommending a home automatic external defibrillator and CPR training for family members [94].

## 7. Multi-Disciplinary Team Approach

**Figure 5:**
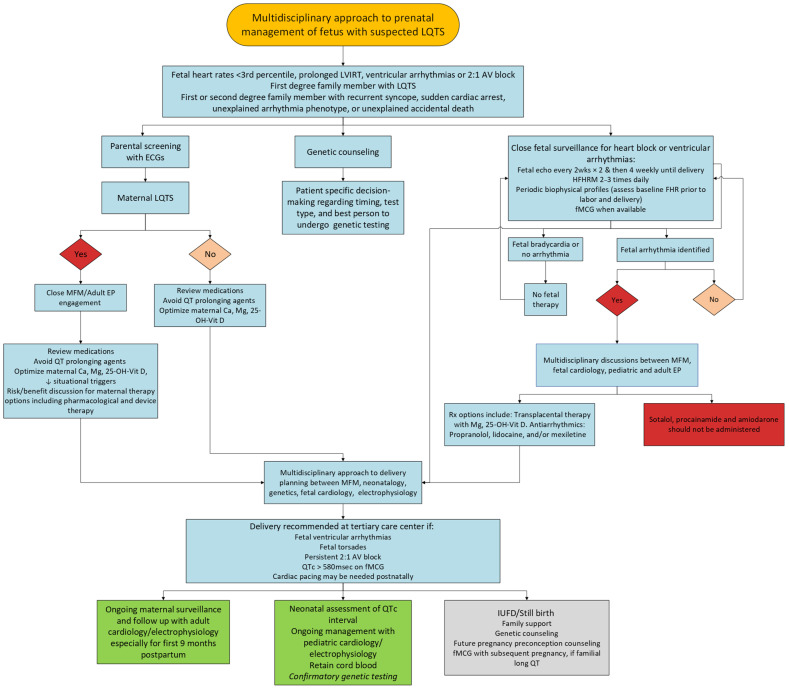
Flow diagram showing multidisciplinary approach to the prenatal management of suspected LQTS from diagnosis through delivery.

As evidenced by the multiple care considerations in the diagnosis and management of LQTS, fetal patients with suspected LQTS require a multidisciplinary care approach with a dedicated team [15,24,95]. Figure 5 shows the details of critical steps and specialists involved from diagnosis through delivery. The fetal cardiologist provides diagnostic testing for the fetus and counseling related to the possibility of LQTS. Genetic counselors review genetic mutations associated with LQTS and options for genetic testing, as well as the coordination of genetic testing based on parental desires. Consultation with pediatric electrophysiologists is helpful for parents to understand treatment options for their child after birth and the anticipated long-term outcomes. Neonatologists are involved in discussions related to the delivery-room management and immediate postnatal care of newborns with suspected LQTS. Pregnancy care is also important and provided by obstetricians or MFM specialists, especially if the pregnant individual has suspected or confirmed LQTS. Lastly, if the pregnant individual is found to have LQTS during this evaluation, they may consult the adult electrophysiology team to discuss her cardiac care.

## 8. Future Directions

Future study is needed to continue improving the diagnosis and management of LQTS in the fetus. Although fMCG has proven to be a reliable diagnostic method to identify prolonged QT interval in utero, it is not widely available at this time. An increased availability of fMCG could improve diagnostic rates, especially as SQUID magnetometers are replaced by new cost-effective optically pumped magnetometer-based sensors that require only “person-sized” shields [96]. Improvements in fetal ECG diagnostic capabilities may provide an alternative to fMCG. The incorporation of speckle tracking echocardiography to assess strain may also aid in the diagnostic capabilities of fetal echocardiography. For example, as compared to normal controls, patients with LQTS demonstrated prolonged left ventricular contraction duration in certain myocardial segments by myocardial strain analysis [97,98]. This modality has become increasingly studied on fetuses of diabetic mothers as well as those with congenital heart disease, and normal fetal ventricular strain values are beginning to be established [99,100,101]. As the use of speckle tracking for fetal ventricular strain increases in the future, differences in these measurements between LQTS and normal fetuses can be studied to assess for similar changes already seen in postnatal imaging.

Genetic testing capabilities have expanded in recent years, and this testing can provide supportive information for fetal diagnosis of LQTS. Increasing the identification of VUS during this testing requires continued follow up by geneticists and genetic counselors to ensure an accurate classification of variants is completed to appropriately provide future reference for care teams. Future pharmacogenomic testing in mother and fetus could aid in understanding the response to beta-blocker therapy for TdP as suggested by Putra et al., but this will require additional study for validation and formal recommendations [102]. Over time, the continued use of genetic testing in the setting of fetal and neonatal presentation, as well as an assessment of the genetics of stillbirths and sudden infant death syndrome cases, should help to bring about a more realistic picture of inherited arrhythmia syndromes in this age range. It will be important to continue to advocate for insurance coverage for genetic counseling, genetic testing, and to improve access to high-level services such as fetal cardiac care teams and fMCG.

## 9. Conclusions

Congenital LQTS presents diagnostic and management challenges that benefit from a multi-disciplinary care team including fetal cardiology, adult electrophysiology, MFM, genetics, neonatology, and pediatric electrophysiology to maximize the care of both the mother and the fetus. Multiple diagnostic modalities including fetal echocardiograms, fMCG, and fetal and familial genetic testing work together to produce the diagnosis and guide management, delivery-room planning, and postnatal care expectations.

## Data Availability

No new data were created or analyzed in this study. Data sharing is not applicable to this article.

## Abbreviations

The following abbreviations are used in this manuscript:

LQTS	Long QT syndrome
ECG	Electrocardiogram
AV	Atrioventricular
BPM	Beats per minute
FHR	Fetal heart rate
fMCG	Fetal magnetocardiography
SQUID	Superconducting quantum interference device
TdP	Torsades de Pointes
FDA	Food and Drug Administration
NIFECG	Noninvasive fetal electrocardiography
fECG	Fetal electrocardiogram
LVIRT	Left ventricular isovolumetric relaxation time
CVS	Chorionic villus sampling
VUS	Variants of uncertain significance
MFM	Maternal–fetal medicine
HFHRM	Home fetal heart rate monitoring
ICD	Implantable cardioverter-defibrillator
